# Effects of reverse deployment of cone-shaped vena cava filter on improvements in hemodynamic performance in vena cava

**DOI:** 10.1186/s12938-021-00855-x

**Published:** 2021-02-09

**Authors:** Ying Chen, Zaipin Xu, Xiaoyan Deng, Shibo Yang, Wenchang Tan, Yubo Fan, Yong Han, Yubin Xing

**Affiliations:** 1 College of Engineering and Technology, Beijing Institute of Economics and Management, Beijing, 100102 China; 2 College of Engineering, Peking University, Beijing, 100871 China; 3 Shenzhen Graduate School, Peking University, Shenzhen, 518055 China; 4 Key Laboratory for Biomechanics and Mechanobiology of Ministry of Education, School of Biological Science and Medical Engineering, Beijing Advanced Innovation Centre for Biomedical Engineering, Beihang University, Beijing, 100083 China; 5 Department of Veterinary Medicine, College of Animal Science, Guizhou University, Guiyang, 550025 Guizhou China; 6 School of Automation and Information Engineering, Sichuan University of Science and Engineering, Zigong, 643002 Sichuan China; 7 Guizhou Institute of Animal Husbandry and Veterinary Science, Guiyang, 550025 Guizhou China; 8 Department of Infection Management and Disease Control, The General Hospital of People’s Liberation Army, Beijing, 100853 China

**Keywords:** Cone-shaped vena cava filter, Hemodynamic performance, Reverse deployment, Computational fluid dynamics

## Abstract

**Background:**

Cone-shaped vena cava filters (VCFs) are widely used to treat venous thromboembolism. However, in the long term, the problem of occlusion persists even after the filter is deployed. A previous study hypothesized that the reverse deployment of a cone-shaped VCFs may prevent filter blockage.

**Methods:**

To explore this hypothesis, a comparative study of the traditional and reverse deployments of VCFs was conducted using a computational fluid dynamics approach. The distribution of wall shear stress (WSS) and shear stress-related parameters were calculated to evaluate the differences in hemodynamic effects between both conditions. In the animal experiment, we reversely deployed a filter in the vena cava of a goat and analyzed the blood clot distribution in the filter.

**Results:**

The numerical simulation showed that the reverse deployment of a VCF resulted in a slightly higher shear rate on the thrombus, and no reductions in the oscillating shear index (OSI) and relative residence time (RRT) on the vessel wall. Comparing the traditional method with the reversely deployed cases, the shear rate values is 16.49 and 16.48 1/s, respectively; the minimal OSI values are 0.01 and 0.04, respectively; in the vicinity of the VCF, the RRT values are both approximately 5 1/Pa; and the WSS is approximately 0.3 Pa for both cases. Therefore, the reverse deployment of cone-shaped filters is not advantageous when compared with the traditional method in terms of local hemodynamics. However, it is effective in capturing thrombi in the short term, as demonstrated via animal experiments. The reversely deployed cone-shaped filter captured the thrombi at its center in the experiments.

**Conclusions:**

Thus, the reverse deployment of cone-shaped filters is not advantageous when compared with the traditional method in terms of local hemodynamics. Therefore, we would not suggest the reverse deployment of the cone-shaped filter in the vena cava to prevent a potentially fatal pulmonary embolism.

## Background

**Fig. 1:**
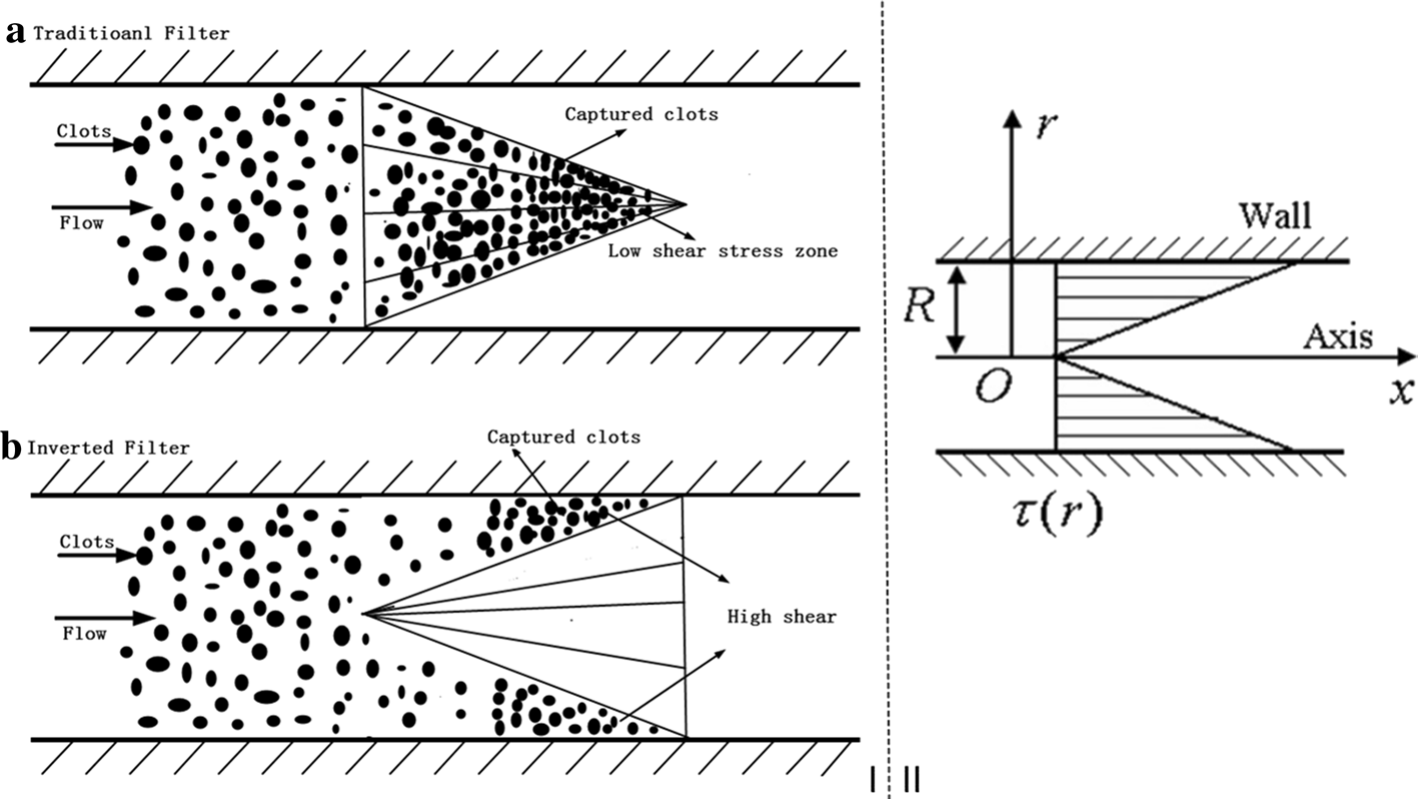
Schematic drawings of traditional and reverse placements of VCF. **I**: **a** traditional deployment; **b** reverse deployment. Note that this is only a diagram and does not represent the specific size of the vena cave or VCF. **II**: Hagen–Poiseuille flow shear-stress distribution. VCF: vena cava filter

Venous thromboembolism (VTE), including deep vein thrombosis (DVT) and pulmonary embolism (PE), is a leading global cause of high morbidity and mortality [1]. Anticoagulants are the first choice for the prevention and treatment of VTE [2]. However, when the use of anticoagulants is contraindicated or VTE recurs despite optimal anticoagulation, vena cava filter (VCF) intervention is recommended to prevent a potentially fatal PE [3, 4]. Commercially available cone-shaped VCFs, such as Option™, Vena Tech, and Greenfield filters, are widely used clinically. However, these filters tend to trap blood clots in their cone-shaped centers [5] (Fig. 1Ia), and the filter blockage problem therefore remains unresolved [6, 7].

To address this issue, a hypothesis of the reverse deployment method for a cone-shaped filter was proposed [8] (Fig. 1Ib). With this hypothesis, in the flow of a circular tube, the flow-induced shear stress distribution is distributed based on Poiseuille’s law as follows:

1 $$\tau (r) = \frac{4Q\mu }{{\pi R^{4} }}r.$$

Here, Q represents the volume flow rate, $\mu$ denotes the viscosity, R denotes the semi-diameter of the tube, $\pi$ denotes the circular constant, and r represents the change in the radial coordinate from 0 of the axis to R of the wall. Therefore, the shear stress at the center evidently is lower than the shear stress in the peripheral area [8]. It is proposed that, by reversing the filter, the blood flow stream would force the blood clots to remain within the peripheral areas of the vena cava, thereby leaving the central passage open, whereas the higher flow-induced wall shear stress (WSS) in the peripheral area can dissolve the captured blood clots. Regions of low shear stress and slow flow can be thrombogenic because of the accumulation of thrombin [9]. High shear stress can enhance the removal of thrombin and fibrin, thereby reducing the likelihood of secondary hemostasis [9]. High levels of shear stress can also stimulate endothelial cells that secrete a tissue plasminogen activator, reducing the risk of hemostasis [10]. Based on this hypothesis, the wall shear stresses (WSSs) acting on the thrombus (center versus peripheral) should be compared. If the WSS on the thrombus deposited at the periphery is higher than that at the center, the thrombus may be dissolved. Shear stress is the component of stress coplanar with the material cross section so that the friction between the blood and thrombus is the key factor. Therefore, this study investigated the hemodynamics of a reversely deployed traditional VCF.

In this study, we designed a numerical study and an animal experiment to assess whether the reverse deployment of a cone-shaped VCF could indeed improve the hemodynamic performance of the filter by inducing high shear stress. As the hypothesis indicates, reverse deployment would induce higher shear stress, which can dissolve the thrombin faster. Therefore, through a numerical study, we compared the reverse deployment method with the conventional method, mainly focusing on the shear stress and shear stress-related parameters, such as the WSS, oscillatory shear index (OSI), and relative residence time (RRT). In the animal experiment, we reversely deployed a cone-shaped filter in the vena cava of a goat and analyzed the blood clot distribution in the filter.

## Results

### Simulation results

#### Shear rate

**Fig. 2:**
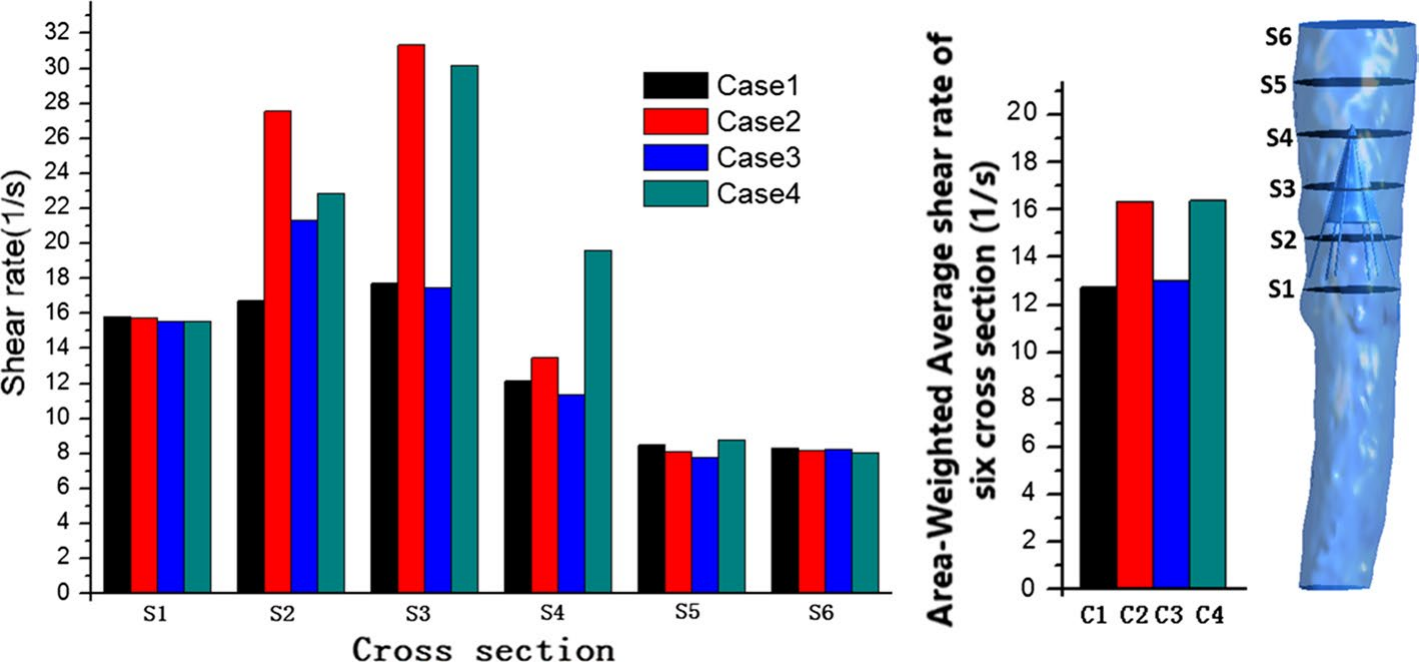
Shear rate for the four cases. Right: six cross sections in the vena cava model, with each adjacent side distance measuring 10 mm. Left: plots of the shear rate and area-weighted average shear rate at six representative cross sections of four cases from steady-flow computations

Several representative cross sections were selected in the vena cava model to compare the average shear rate in the different cases, and the area-weighted average shear rates for six representative cross sections were plotted (S1–S6, Fig. 2).

**Fig. 3:**
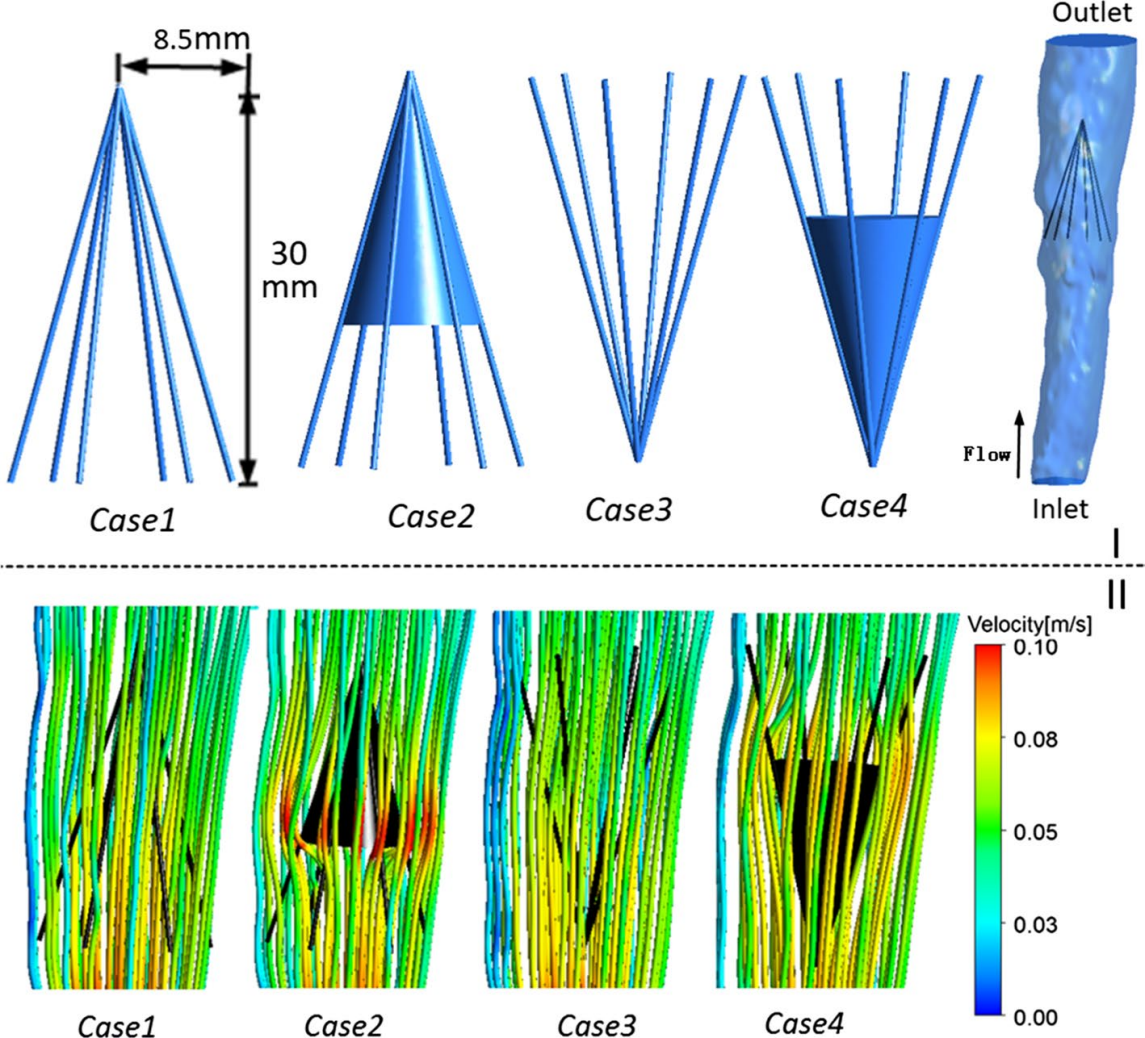
Models, inlet waveform velocity, and streamlines. **I**: Model diagrams of the four cases with and without thrombi. The diagram also shows the cone-shaped filter in the vena cava used in the computations. **II**: peak systole (time = *t*2) velocity streamline for the four cases from pulsatile flow computations

Figure 2 illustrates the shear rate profiles of the six representative cross sections of the vena cava based on different cases. The shear rate of the blood flow adjacent to the filters exceeded that in other regions within the vena cava in all models. Near the filter, Cases 2 and 4 clearly induced higher shear rates with thrombi from S2 to S4; the maximum shear rates for Cases 2 and 4 are shown in S3. By comparison, the shear rates for Cases 1 and 3, without thrombi, were lower. In S5 and S6, which are located far from the filter and thrombi, no evident difference between the different cases was found.

#### Flow pattern

Figure 3II presents the peak systole (time = *t*2) velocity streamlines for the four cases colored by the velocity magnitude. In Cases 2 and 4 with thrombi, the velocity clearly increased near the filter and thrombus. For Case 2, at the bottom of the cone-shaped thrombus, the streamline was clearly red, which represents a high velocity. The streamlines are smoother than those in Cases 1 and 3 without a thrombus.

**Fig. 4:**
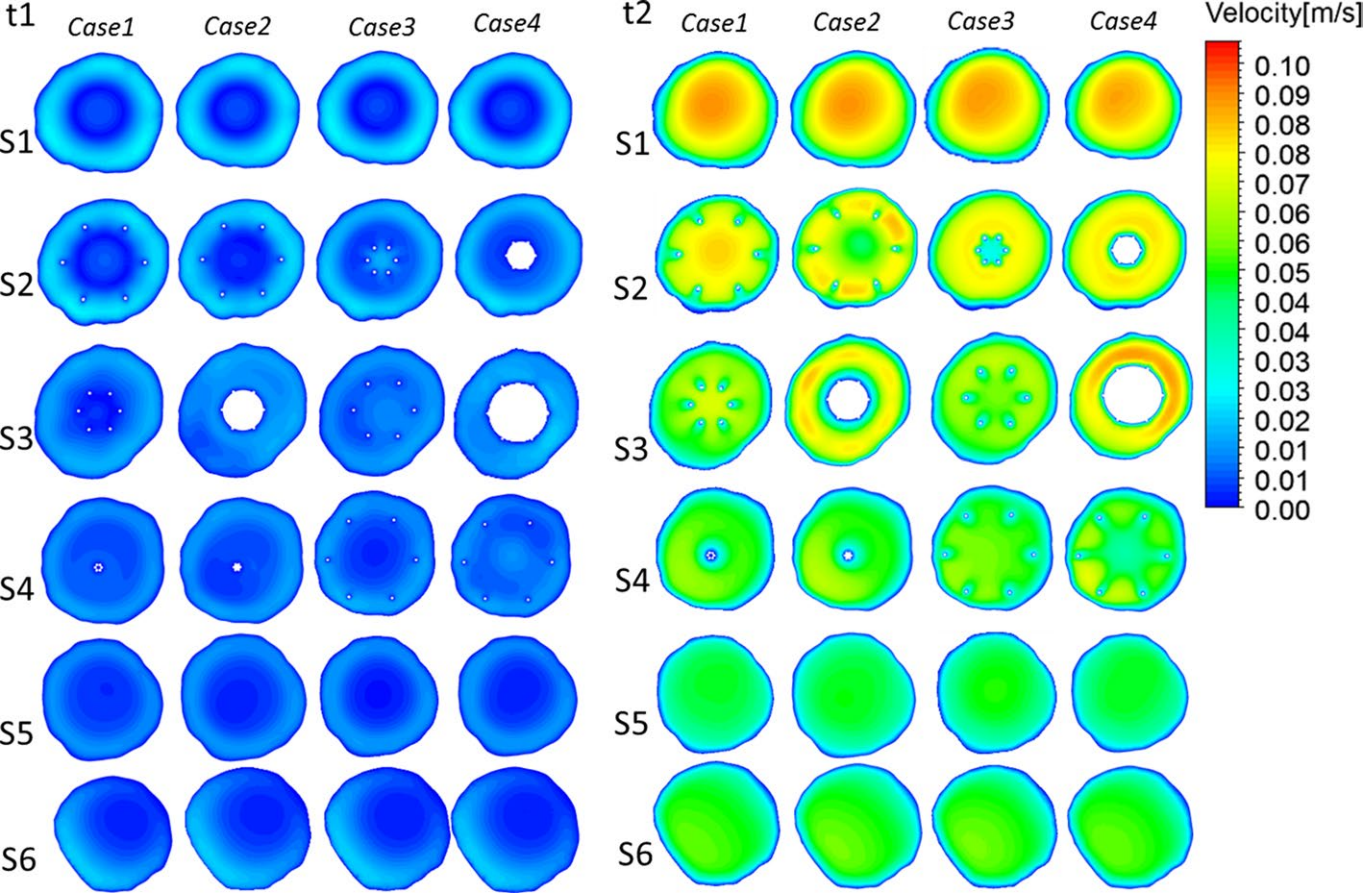
Velocity distributions from pulsatile flow computations. Time = *t*1 and time = *t*2 velocity distributions from pulsatile flow computations

**Fig. 5:**
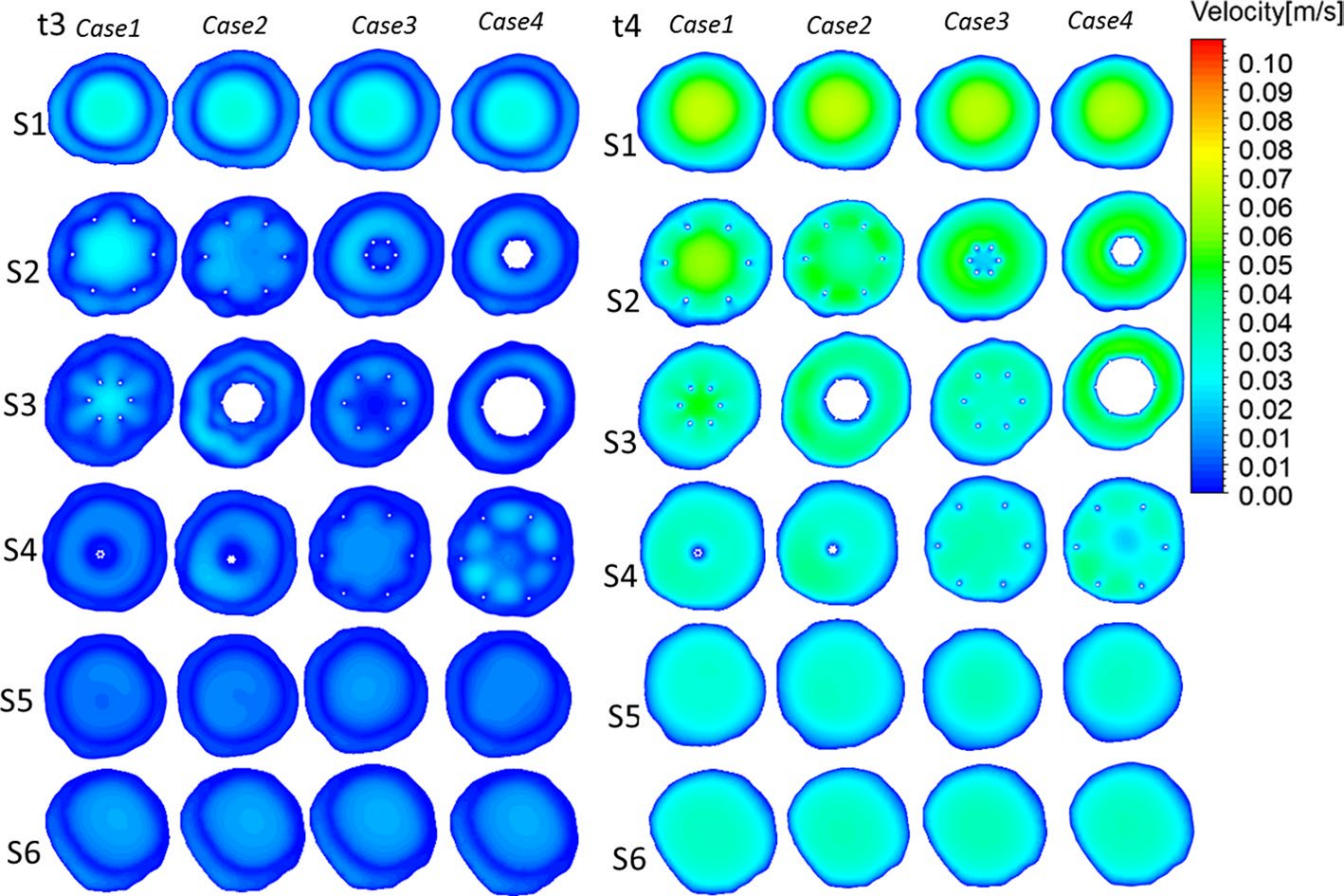
Velocity distributions from pulsatile flow computations. Time = *t*3 and time = *t*4 velocity distributions from pulsatile flow computations

Figures 4 and 5 present the velocity profiles within the six planes of each vena cava during the cardiac cycle. Notably, the cutting planes (S1–S6 in Fig. 2) represent the same positions for all cases. Because the cutting plane does not present the same position, the velocity contours must be different. For instance, S3 is at approximately the middle of the thrombus in Case 4, whereas S2 is at approximately the middle of the thrombi in Case 2.

**Fig. 6:**
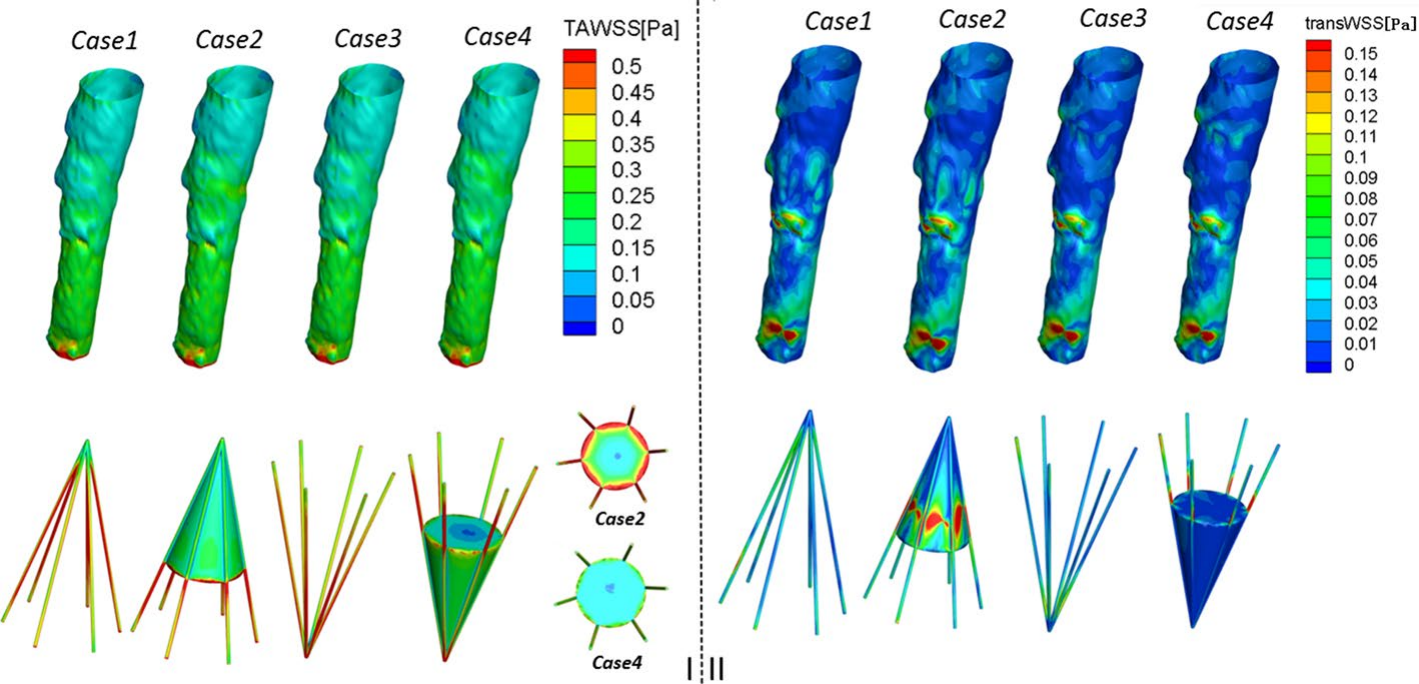
Contours of TAWSS and transverse WSS from pulsatile flow computations. **I**: contours of TAWSS on the caval wall and filter. **II**: contours of transverse TAWSS on the caval wall and filter. TAWSS: time-averaged wall shear stress

In general, among all the cases considered, excepting that of the inner filter, none of the models significantly changed the blood flow velocity within the vena cava during the overall cardiac cycle. These results are consistent with those obtained in a previous study by Singer et al., who reported that different thrombi shapes generally result in similar shear stresses and velocity profiles when thrombi are trapped within the central inferior vena cava [11]. However, the increase in the flow velocity in the periphery of a filter is more pronounced because of the existence of a thrombus. For example, in S3 of both Cases 2 and 4, the velocity in the periphery of the thrombi is evidently higher. In conclusion, in Cases 2 and 4, the presence of thrombi leads to higher velocities along their peripheries.

#### Time-averaged and transverse WSS on the vessel wall and filter/thrombus

The time-averaged wall shear stress (TAWSS) contours of the vessel models in the four cases are shown in Fig. 6I. The TAWSSs on the vessel wall for these cases are qualitatively similar. Specifically, in the vicinity of the filter, the TAWSS on the vessel wall increases with the existence of thrombi. In Cases 3 and 4, in which the filters are reversely deployed, the TAWSS of the cone vertex of the filter is slightly higher than in Cases 1 and 2, in which the filters are traditionally deployed. As shown in Fig. 6I, the TAWSS on the filter is extremely high in the absence of a thrombus. When thrombi are present in the center of the filter, the TAWSS of Case 2 is evidently higher than the TAWSS of Case 4, particularly at the bottom of the thrombus cone. Furthermore, Fig. 6I also shows that the TAWSS on the filter decreases after thrombus deposition, particularly in the joint region between the struts and thrombus.

**Fig. 7:**
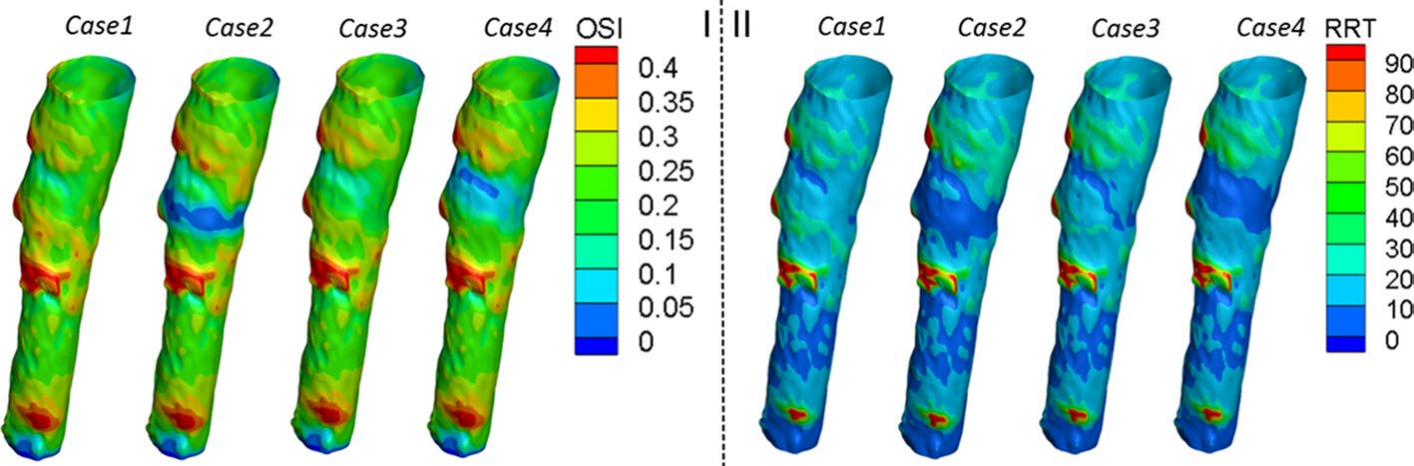
OSI and RRT for the four cases. **I**: OSI on the caval wall. **II**: RRT on the caval wall. OSI: oscillatory shear index; RRT: relative residence time

Figure 6II shows the transverse WSS distribution on both the vessel wall and filter. In general, similarly to the TAWSS distribution, the transverse WSS on the thrombus in the traditionally deployed cases is higher than that in the reverse deployment cases. In Case 2, in particular, the transverse WSS on the surface of the thrombi is shown in red and thus has a high value

#### Oscillatory shear index and relative residence time

As shown in Fig. 7, the OSI weakens after thrombi deposition and is more evident within the vicinity of the filter/thrombus. When compared with Cases 2 and 4, the reverse deployment case leads to a slightly higher OSI than the traditional deployment case within the thrombus area. Specifically, in Cases 2 and 4, a lower OSI location appeared in this vicinity.

**Fig. 8:**
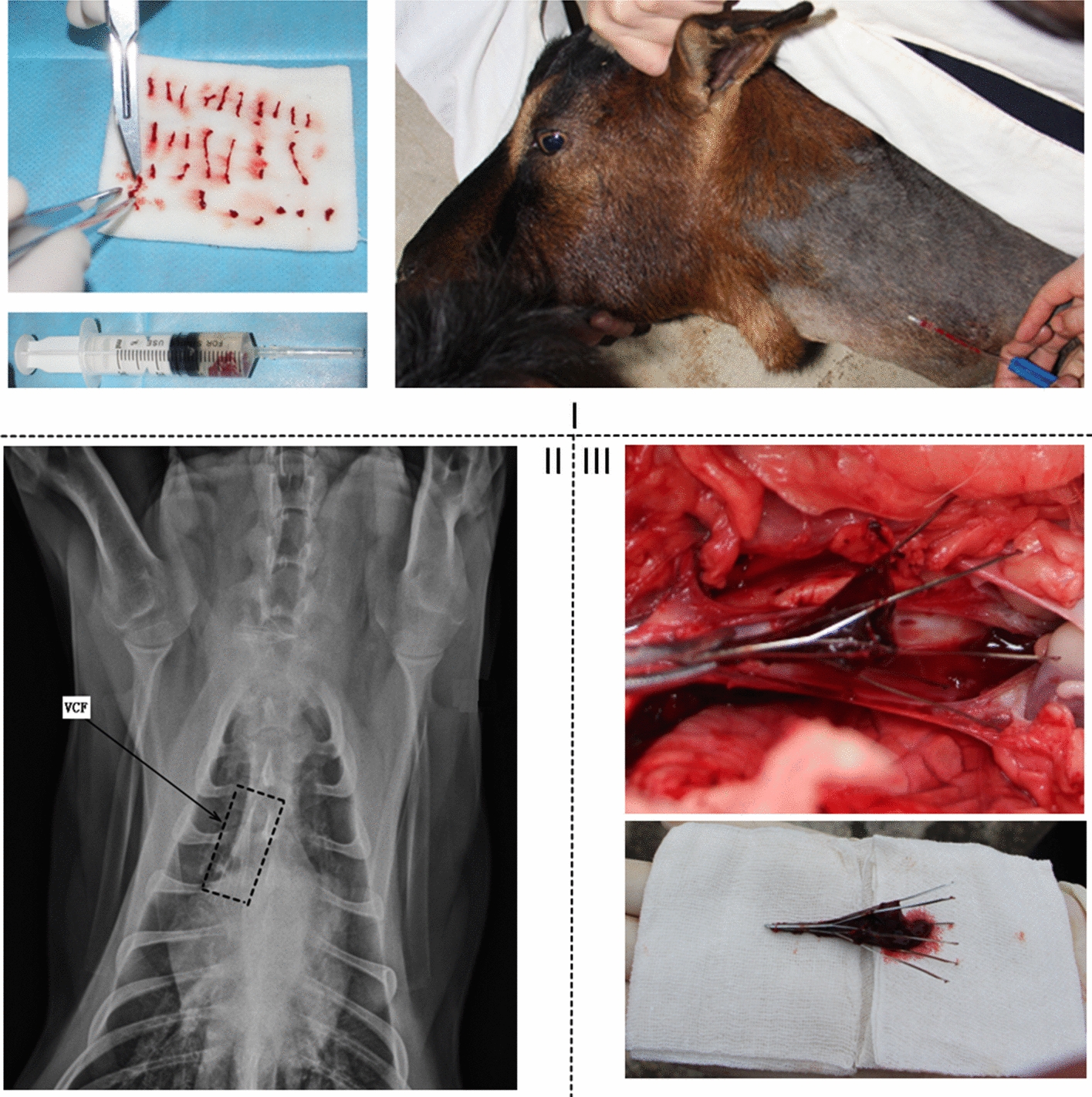
Autologous blood clots are injected into the left jugular vein of the goat; CR image; experimental results. **I**: cylindrical thrombus on the sterile gauze and mixed solution of autologous thrombus. A reverse cone-shaped filter with a mixed solution of blood clots was injected into the jugular vein. **II**: CR image display indicates showing the VCF explanted in the superior vena cava of the goat. **III**: experimental results: reversed cone-shaped filter deposited thrombus. CR: computed radiography; VCF: vena cava filter

Figure 7 also clearly demonstrates that the existence of thrombi decreases the RRT on the vessel wall, particularly in the vicinity of the thrombi. A comparison of no-thrombus Case 1 and thrombus Case 2 shows that the RRT on the vessel wall decreases from 20 to 10 within the thrombus area.

#### Animal experimental results

After euthanizing the goat, an autopsy was immediately conducted to observe the filter. It was observed that the filter was explanted in the superior vena cava without tilting. The diameter of the superior vena cava was 15.2 mm, and the end of the filter was near the heart. Notably, the thrombi continued to be deposited within the center of the filter with the reverse deployment of the cone-shaped filter in the vena cava of the goat (Fig. 8III). There were no visible injuries such as a vascular intima, vessel wall fracture, or adhesion at the hooked site. The surface of the lung did not exhibit any abnormalities or embolism.

**Fig. 9:**
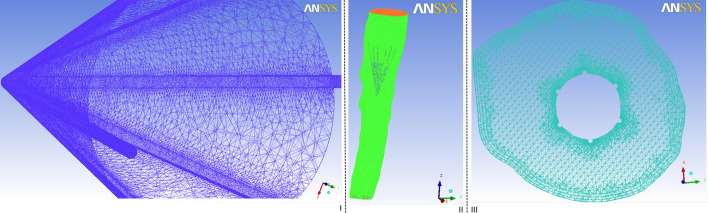
Mesh distribution for Case 4. **I**: mesh distribution around the filter and thrombus. **II**: overall view of the structure. **III**: plane mesh distribution around the thrombus

### Discussion

VCF occlusion after filter deployment continues to be a challenge even in the modern era of medicine. This study investigated the use of a VCF with reverse deployment to determine whether it improved the local hemodynamic performance in a vena cava. This was accomplished by conducting numerical simulations and an in vivo experiment.

The motion of blood in a straight vessel under a pressure gradient balances the energy loss resulting from friction. The fluid velocity is higher at the center, and blood clots may be easily deposited in the peripheral areas of the vessel. Therefore, prior to conducting the animal experiment, it was expected that the thrombus would be trapped near the vessel wall. However, the experimental results showed that the thrombus was still deposited at the center of the filter. At a broader level, it is inferred that the flow of blood in the vena cava does not correspond to a standard circular tube flow. The direction of blood flow in the vena cava constitutes a major backflow from the jugular vein to the heart. However, the velocity of blood in the vena cava fluctuates, and this clearly differentiates it from the Poiseuille flow in a simple straight circular tube. According to the previous hypothesis, high shear stress may dissolve the thrombin more rapidly [10]; the accumulation of clots at the center of the filter is poor because the clots slowly dissolve in a low-shear flow. Therefore, the results of the animal experiment showed that this hypothesis does not hold for the in vivo vena cava.

The results also showed that the new deployment method did not improve the hemodynamics of the caval wall in terms of the TAWSS or transWSS when compared with those of a traditional method. The simulation results also concluded that with the exception of the cone vertex, the use of reverse deployment decreased the TAWSS and transWSS on the surface of the thrombi. The geometry of the inverted thrombi favored the development of blood stasis because of the lower WSS at the bottom of the thrombi. A low WSS is usually associated with blood flow stasis and thrombosis [12]. Furthermore, the reverse deployment also leads to a slightly higher OSI and RRT within the vicinity of the filter. Low WSS and high OSI values are important factors in intimal hyperplasia, because they affect the function of endothelial cells [13]. A high OSI and RRT can lead to thrombus formation by simulating platelet aggregation, enhancing the collisions of the activated platelets, and increasing the residence time of procoagulant microparticles [14]. Consequently, a reverse deployment can act adversely in dissolving the deposited thrombi, thereby further increasing the risk of PE. Furthermore, as a potential disadvantage of the inverted cone-shaped filter, clots propelled to the periphery of a reversed filter are more likely to pass through and become PEs. These thrombi attached to the downstream side of the filter are more susceptible to embolization than those captured in the filter placed in a traditional orientation.

To date, there is still no solid evidence showing the significance of the hemodynamics in thrombus formation at the center or on the wall at the early formation stage. The thrombus formed on the filter may be primarily because of the structure of the filter. As aforementioned, the maximum blood velocity occurs along the center of the vessel, and the minimum velocity appears near the wall. Therefore, thrombi are mostly distributed at the center of the bloodstream. The struts are densely distributed at the center of the filter regardless of how the filter is deployed, resulting in thrombi being easily attached to the struts. In the long term, after a thrombus is formed on a reversely deployed filter, vortices may be generated on the backside of the clot owing to fluid dynamics, potentially causing an accumulation of the clot. In the future, we would like to conduct such a study, including more animal experiments to determine whether the strut of the filter can hold the clot when the thrombus grows, or whether it leads to a risk of shedding, thereby blocking the downstream vessels and the heart.

As a preliminary consideration, this study has certain limitations. First, we assumed the thrombus shape as a standard cone volume in our numerical simulation. An extant study showed that no single thrombus shape can be rigorously assigned to a clot owing to the inherently complex and random nature of its formation [15]. However, according to the findings reported by Wang, spherical-shaped and conical-shaped thrombi are the most representative in clinical patients [16]. We also considered the results from the animal experiment, which revealed that the captured thrombus was cone-shaped. Therefore, we finally modeled the thrombus as a cone shape in the simulation study. Second, owing to the high costs involved in conducting an in vivo experiment, the outcome of our experiment outcome is for a single animal. Third, we assumed a velocity profile with an axial velocity component and a transverse velocity component equal to zero at the inlet. For a venous flow, the flow velocity is low relative to that in the arteries. As part of future investigations, we intend to consider the pulsatile velocity profile even though a parabolic velocity profile was assumed. An optional solution is to extend the length of the inlet to provide a sufficient path and allow the numerical approach to generate the velocity profile based on the pulsatile velocity waveform. In addition, there are still other limitations of the simulation study, including single vessel geometry, a rigid wall used in the simulations, and a parabolic velocity profile instead of a Womersley velocity profile. All of these aspects should be addressed in future studies.

## Conclusions

In conclusion, the results of this study showed that the reverse deployment method is no better than the traditional deployment in terms of the biomarkers from numerical simulations. Therefore, based on the results of the numerical simulations and a single in vivo experiment, we would not suggest the reverse deployment of the cone-shaped filter in the vena cava to prevent a potentially fatal PE.

## Methods

### Simulation study methods

#### Geometric model and meshing

Utilizing a prototype of a Greenfield VCF [3, 17], and vena cava structure sizes extracted from [18], we used Pro/Engineer (version Wildfire 4.0, Parametric Technology Co., USA) and created a cone-shaped filter. The cone-shaped filter was simplified into six symmetrical circular struts each, with a diameter of 0.5 mm. The length of the filter was 30 mm. Figure 3I shows four cases that represent a traditional deployment and a reverse deployment with or without a thrombus, which were investigated using numerical simulations. The volume of the cone thrombus used in the numerical simulation was 0.565 cm^3^, which is consistent with the volume reported by Wang et al. [16, 19]. The vena cava model, as shown in Fig. 3I, was reconstructed based on computed tomographic images from a healthy male volunteer aged 58 years. The volunteer provided written informed consent to this study, which was approved by the Ethical Committee of the General Hospital of the People’s Liberation Army and carried out in accordance with the regulations of the hospital. We reconstructed the vena cava model images using the commercial three-dimensional reconstruction software Mimics (Materialize, Belgium). Next, Geomagic (Geomagic, USA) was used to improve the quality of the model surface. The vena cava model was 109 mm long.

In each case, all computational models were meshed using tetrahedral and hexahedral elements using ANSYS ICEM (ANSYS Inc., Canonsburg, PA). To ensure that the results were mesh-independent and validated, a grid-adaptation technique was used, which refined the grid based on the geometric and numerical solution data. Boundary layers near the vessel wall were also applied, which were set to 3; the height ratio was set to 1.2, and the total height was set to 0.2 mm. High-density mesh elements were applied close to filters, and the maximum size of the filter was set to 0.15 mm. The final volumes of the meshes corresponded to 3 298 245, 3 838 665, 3 318 325, and 3 798 220 for Case 1, 2, 3, and 4, respectively. In particular, the mesh distribution for Case 4 is presented in Fig. 9.

**Fig. 10:**
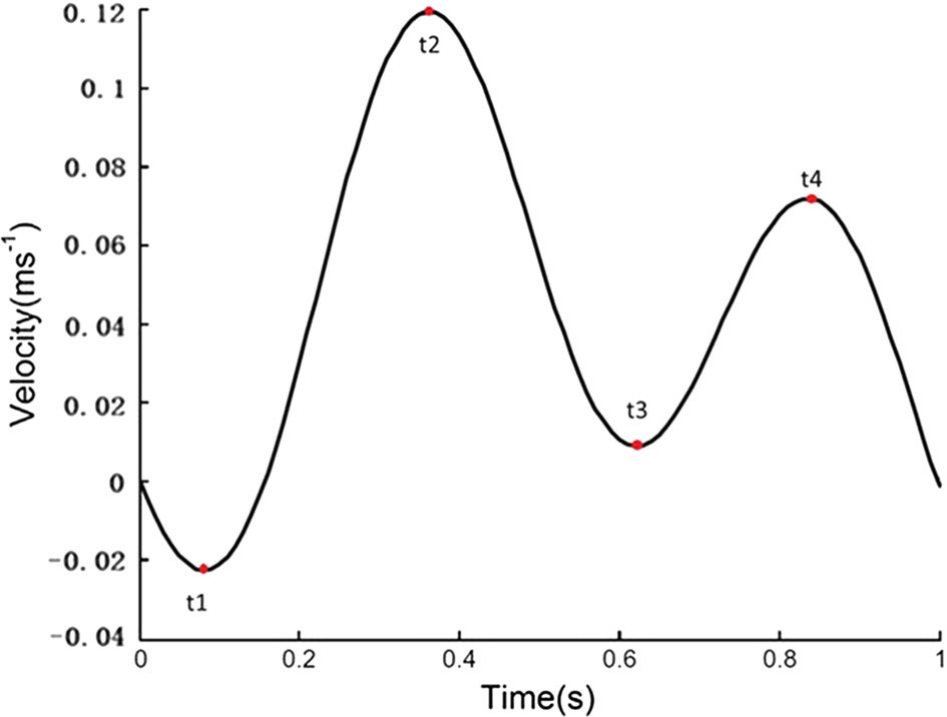
Inlet inferior vena cava waveform velocity used in the pulsatile flow computations

#### Assumptions

In this study, simulations were performed assuming laminar flow conditions [20]. Blood was assumed to be a homogeneous and incompressible non-Newtonian fluid. Notably, our previous study showed a similar difference in the flow features between the Newtonian numerical simulations and Carreau model simulations in the vena cava [21]. Therefore, only the Carreau model simulation results are provided in this study.

#### Governing equations

Flow simulations were performed based on the three-dimensional, incompressible Navier–Stokes and the continuity equations as follows [22]:

2 $$\rho ((\partial \upsilon /\partial t) + (\upsilon \cdot \nabla )\upsilon ) = - \nabla p + \nabla \cdot \tau ,$$

3 $$\nabla \cdot \upsilon = 0,$$

where $\upsilon$ and *p* are the fluid velocity vector and pressure, respectively; *ρ* = 1050 kg/m^3^ is the blood density; and $\tau$ is the tension tensor, which is expressed as follows:

4 $$\tau = 2\eta (\dot{\gamma })D.$$

Here, *D* and $\dot{\gamma }$ are the respective deformation rate tensor and shear rate, respectively, and $\eta$ is the viscosity, which is a function of the shear rate.

The Carreau model is used to calculate the viscosity of blood as follows:

5 $$\eta (\dot{\gamma }) = \eta_{\infty } + (\eta_{0} - \eta_{\infty } )[1 + (\lambda \dot{\gamma })^{2} ]^{((n - 1)/2)} ,$$

where $\eta_{\infty }$ = 3.45 $\times$ 10^−3^ kg/(m s), $\eta_{0}$ = 5.6 $\times$ 10^–2^ kg/(m s), *n* = 0.3568, and $\lambda$ = 3.313 s [23].

#### Hemodynamic parameters

The shear stress on the vessel wall throughout a cardiac cycle was evaluated by using the TAWSS, which is expressed as follows:

6 $$\mathrm{TAWSS}=\frac{1}{\mathrm{T}}{\int }_{0}^{\mathrm{T}}\left|\mathrm{WSS}\left(\mathrm{s},\mathrm{t}\right)\right|\mathrm{dt},$$

where *T* is the cardiac cycle period, WSS is the instantaneous wall shear stress vector, and s is the position on the caval wall. The OSI indicates the changing frequency of the wall shear-stress direction as follows [24]:

7 $$\mathrm{OSI}=\frac{1}{2}\left[1-\left(\frac{\frac{1}{\mathrm{T}}\left|{\int }_{0}^{\mathrm{T}}\mathrm{WSS}\left(\mathrm{s},\mathrm{t}\right)\cdot \mathrm{dt}\right|}{\frac{1}{\mathrm{T}}{\int }_{0}^{\mathrm{T}}\left|\mathrm{WSS}\left(\mathrm{s},\mathrm{t}\right)\right|\mathrm{dt}}\right)\right],$$

$$0\le \mathrm{OSI}\le \frac{1}{2}.$$

A zero OSI value corresponds to a unidirectional shear flow, and the OSI value is 1/2 when a purely oscillatory shear case occurs [25].

Another useful parameter, the RRT, was also calculated. Specifically, RRT reflects the residence time of flow particles near the caval wall, and it is also recommended as a single metric of low and oscillating shear stress [26]. Thus, RRT is defined as follows [27]:

8 $$\mathrm{RRT}=\frac{1}{\left(1-2\cdot \mathrm{OSI}\right)\cdot \mathrm{TAWSS}}.$$

The OSI does not distinguish well between uniaxial pulsatile flow and multidirectional flow. Therefore, the parameter, namely, transWSS, was also introduced. It is expressed as follows [28]:

9 $$\mathrm{transWSS}=\frac{1}{\mathrm{T}}{\int }_{0}^{\mathrm{T}}\left|\mathrm{WSS}\cdot \left[n\times \frac{\frac{1}{\mathrm{T}}{\int }_{0}^{\mathrm{T}}\mathrm{WSS}\left(\mathrm{s},\mathrm{t}\right)\cdot \mathrm{dt}}{\left|\frac{1}{\mathrm{T}}{\int }_{0}^{\mathrm{T}}\mathrm{WSS}\left(\mathrm{s},\mathrm{t}\right)\cdot \mathrm{dt}\right|}\right]\right|\mathrm{dt},$$

where *n* is normal to the vessel surface. This new metric has clear advantages over other parameters that have attempted to capture multidirectional aspects. It also appears to be more sensitive to changes in the velocity waveform [29]. Thus, it complements TAWSS and OSI as opposed to replacing them.

#### Boundary conditions and computation

In all cases, a steady-flow simulation was first performed. The solution obtained from this simulation was then used as the initial iteration data for further pulsatile flow simulations. With respect to the steady-flow simulation, a uniform inflow velocity profile with an axial velocity component of 0.1 m/s and a transverse velocity component equal to zero were used at the inlet [30]. We set the outlet as the outflow. The caval wall was assumed to be rigid and non-slippery.

For the pulsatile flow simulation, the time-dependent parabolic flow velocity waveform based on the measurement performed by Zhang et al. (as shown in Fig. 10) was set at the inlet [31]. The other boundary conditions were identical to those in the steady computation.

The finite volume method was adopted to solve the mass and momentum conservation equations using ANSYS Fluent 14.0 computational fluid dynamics solver (ANSYS Inc., Canonsburg, PA). The residual continuity and velocity were assigned a value of 1.0 × 10^–5^. Six cycles were required to obtain convergence for the transient analysis, with 200 steps in each cycle (time = 1 s). The pulsatile calculation was performed on a computer equipped with a 2.20 GHz Intel(R) Xeon(R) CPU processor and 64 GB of random access memory (RAM). The computational time-span approached a week for each scenario.

### Animal experiment methods

#### Animal experimental setup

The use of animals in the present study was approved by the local ethical committee (Guizhou Institute of Animal Husbandry and Veterinary Science, Guiyang, China) and was based on the laboratory animal administration rules of China. A healthy adult male goat weighing 51.2 kg was used for the experiment. The goat was anesthetized through an intramuscular injection of a general anesthesia agent of QMB (a product of the Department of Veterinary Surgery, Northeast Agricultural University, China.) at 0.1 mL/kg body weight. Initially, an attempt was made to deploy the VCF in an inferior vena cava from the femoral vein. However, the results indicate that the femoral vein of the goat was excessively thin to allow it to separate from the vessels, thereby making it difficult to insert the filter. The filter was deployed in the superior vena cava from the jugular vein of the goat.

Option™ VCF, a commercially available filter, is cone-shaped and in wide clinical use. In this study, the Option™ VCF (Argon Medical Devices, Frisco, Texas, USA) was inserted into the superior vena cava via the jugular vein of the goat using B-mode ultrasound guidance.

The goat did not exhibit any abnormalities under the preoperative B-mode ultrasound guidance. After anesthesia, the goat was placed in a left lateral decubitus position. The left jugular vein of the goat was fixed. The skin at the surgical site was disinfected as per the standard. The jugular vein was approached using a longitudinal incision in the middle of the neck with adequate vessel separation, and the vessel was subsequently punctured using a puncture needle. The Option™ VCF was then deployed in the superior vena cava according to the routine procedure of VCF deployment.

After deployment, computed radiography (CR; Carestream Vita CR System, Rui Ke Medical Shanghai Co., Ltd.) was used to determine the VCF location. The CR image display showed that VCF was present in the superior vena cava (Fig. 8II).

Eleven days after the VCF deployment, 10 mL off-venous blood was drawn from the goat with the help of a syringe. The blood sample in the syringe was maintained for 30 min at room temperature, and the syringe was then depressed to form cylindrical thrombi on sterile gauze. A cylindrical thrombus of 2–6 mm in length was selected. Autologous thrombi were flushed using physiological saline. Subsequently, a mixed suspension containing at least three clots per 1 mL of physiological saline was prepared. Next, 20 mL of the mixed solution of autologous thrombi was injected into the left jugular vein of the goat (Fig. 8I). Although collagen proteins or other coagulation factors could also simulate a thrombus, these invaders may cause an immune response [32]. Therefore, autologous blood clots are more favorable than other coagulation factors. To develop a canine model of an acuter PE model, extant studies have also considered using autologous blood clots as simulated thrombin [33]. Previous studies revealed that placing a filter in the vena cava for longer than 2 weeks leads to intimal hyperplasia and vascular adhesion [34, 35]. Therefore, 14 days after the VCF deployment, the goat was euthanized through an overdose of anesthesia, and the filter was removed to observe thrombosis capture on the spot.

## Data Availability

All data generated or analyzed during this study are included in this published article.

## Abbreviations

VTE: Venous thromboembolism
DVT: Deep vein thrombosis
PE: Pulmonary embolism
VCF: Vena cava filter
TAWSS: Time-averaged wall shear stress
OSI: Oscillatory shear index
RRT: Relative residence time
WSS: Wall shear stress
CFD: Computational fluid dynamic
CR: Computed radiography
RAM: Random access memory
